# From commensalism to parasitism within a genus-level clade of barnacles

**DOI:** 10.1098/rsbl.2022.0550

**Published:** 2023-07-05

**Authors:** Hiromi Kayama Watanabe, Daisuke Uyeno, Luna Yamamori, Naoto Jimi, Chong Chen

**Affiliations:** ^1^ X-STAR, Japan Agency for Marine-Earth Science and Technology (JAMSTEC), 2-15 Natsushima-cho, Yokosuka, Kanagawa 237-0061, Japan; ^2^ Graduate School of Science and Engineering, Kagoshima University, 1-21-35 Korimoto, Kagoshima 890-0065, Japan; ^3^ Seto Marine Biological Laboratory, Field Science Education and Research Center, Kyoto University, 459 Shirahama, Nishimuro, Wakayama 649-2211, Japan; ^4^ Sugashima Marine Biological Laboratory, Graduate School of Science, Nagoya University, 429-63 Sugashima-cho, Toba, Mie 517-0004, Japan

**Keywords:** cirripedia, convergence, *Octolasmis*, mesoparasite, *Rhizolepas*

## Abstract

Understanding how animals evolve to become parasites is key to unravelling how biodiversity is generated as a whole, as parasites could account for half of all species richness. Two significant impediments to this are that parasites fossilize poorly and that they retain few clear shared morphological features with non-parasitic relatives. Barnacles include some of the most astonishingly adapted parasites with the adult body reduced to just a network of tubes plus an external reproductive body, but how they originated from the sessile, filter-feeding form is still a mystery. Here, we present compelling molecular evidence that the exceedingly rare scale-worm parasite barnacle *Rhizolepas* is positioned within a clade comprising species currently assigned to *Octolasmis*, a genus exclusively commensal with at least six different phyla of animals. Our results imply that species in this genus-level clade represent an array of species at various transitional stages from free-living to parasitic in terms of plate reduction and host-parasite intimacy. Diverging only about 19.15 million years ago, the route to parasitism in *Rhizolepas* was associated with rapid modifications in anatomy, a pattern that was likely true for many other parasitic lineages.

## 1. Introduction

Understanding how parasitism arises from a free-living state is of great importance to unravelling the evolution of animal diversity, as parasites may account for half of it [1]. Though recent genomic studies have shed some light on this [2], due to exceedingly few living taxa at intermediate evolutionary stages the structural route towards parasitism remains poorly understood. Fully parasitic groups, like myxozoan cnidarians, minimized to being among the smallest of animals, the eulimid snail *Enteroxenos* reduced to no more than a pod of gonads [3], and rhizocephalan barnacles transformed into a network of tubules inside their crustacean hosts [4], which usually do not retain morphological similarities with their close relatives at parasitic stages. This poses a significant problem in reconstructing the step-wise adaptation towards parasitism. Cirripedes can potentially provide key insights on this as thoracicalcarean barnacles, sister-clade to the completely parasitic rhizocephalan barnacles, are typically suspension-feeders but also include multiple parasitic groups converging on a rhizocephalan-like body plan [4]. Despite this, the rarity, and a consequent lack of molecular data, of the parasitic forms has kept their evolutionary scenarios largely veiled since Darwin first studied them [5].

*Rhizolepas* is a mesoparasitic barnacle infecting scale-worms, and is characterized by lacking an open mouth or anus as well as possessing an extensive ‘root’ embedded within the host through which it obtains nutrition [6]. This distinctive morphology led to the establishment of the family Rhizolepadidae. The only other thoracican barnacle with comparable morphology is the shark barnacle *Anelasma* [7], famed for first sparking Darwin's interest in barnacles. Originally placed in its own family Anelasmatidae, molecular phylogenetic analysis has revealed *Anelasma* to be a derived pollicipedid closely related to the suspension-feeding intertidal barnacle *Capitulum*, transitioning to parasitism around 120 million years ago (mya) [7,8]. The divergent morphology between *Anelasma* and *Capitulum* highlights the limited capacity of morphology in assessing evolutionary relationships of parasitic taxa [7,9], casting doubt on the positions of other enigmas like *Rhizolepas*. Furthermore, since no intermediates between *Anelasma* and *Capitulum* are known, limited information on transitional stages is available.

With only two species known [10] and each from a single sampling event, the rarity of *Rhizolepas* meant for decades no material was available for sequencing. Recently, we collected an undescribed *Rhizolepas* species attached to the parapodia of an also undescribed *Laetomonice* scale-worm (Aphroditidae) from Kagoshima, Japan. Here, we reveal a surprising phylogenetic position for the thoracican parasitic barnacle *Rhizolepas*, nested within the gooseneck barnacle genus *Octolasmis* previously thought to be in a separate family. This placement coincided with the gall-forming *Rugilepas* infesting sea urchins [11], which we confirm in our phylogeny. We propose that *Octolasmis* represents a lineage showing exceptional adaptations to multiple host phyla, providing important insights into how parasites evolved from free-living ancestors.

## 2. Material and methods

### (a) Sampling

**Figure 1.**
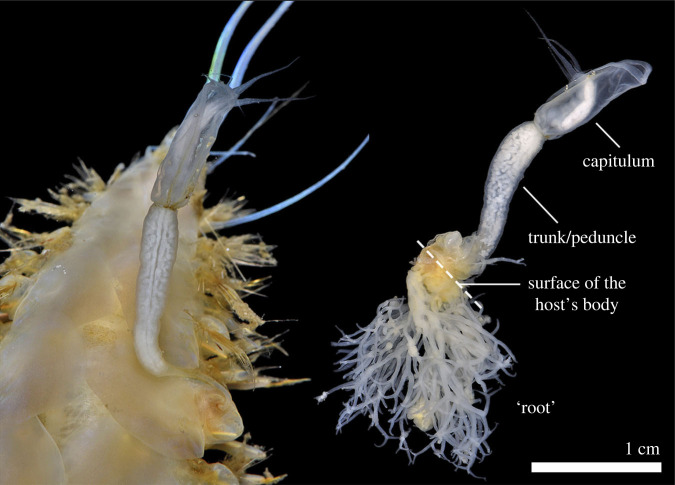
*Rhizolepas* sp. from the scale-worm *Laetomonice* sp. shown in its living posture (left) and a dissected individual showing the root-like structure embedded within the host's tissue (right).

Aphroditid scale worms (*Laetomonice* sp.) parasitized by *Rhizolepas* sp. were collected by bottom trawls from a depth of *ca* 300 m off Kagoshima, southern Japan (31°35.038′N, 129°54.932′E). Approximately one in 10 *Laetomonice* scale-worms were infested by *Rhizolepas* sp. Specimens of *Rhizolepas* sp. (figure 1) were removed from the host worms using a surgical knife, preserved initially in 80% ethanol, and then transferred to 99% ethanol for long-term preservation. For morphological observation, a specimen was dissected by a sharpened tungsten needle under a biological microscope (Leica M80).

### (b) DNA extraction and sequencing

Total DNA was extracted from an egg of an ovigerous *Rhizolepas* sp. individual. The GeneReleaser (BioVentures, Inc.) reagent was used to obtain 30 µl of DNA solution through protocols outlined in [12] but using 25 µl of GeneReleaser and 25 µl of PCR buffer. The partial sequences of 28S rRNA (approx. 1800 bp), 18S rRNA (approx. 1800 bp), 16S rRNA (approx. 400 bp), and cytochrome oxidase *c* subunit I (COI: approx. 650 bp) genes were amplified using the Premix ExTaq Kit (Takara Bio, Inc.) and the primer sets used in previous studies [7,13] by a Veriti Thermal Cycler (Applied Biosystems, ThermoFisher). For details of primers used and their annealing temperatures see electronic supplementary material, table S1. The partial 16S rRNA sequence of *Rugilepas pearsei* was amplified based on the DNA extracted from the same specimen analysed in [11] taken from a coral reef off Manzamo, Okinawa Prefecture, Japan in May 2018 on the sea urchin *Echinothrix diadema*. The amplified DNA fragments were confirmed by 1.0% agarose-gel electrophoresis with RedSafe nucleic acid staining solution (iNtRON Biotechnology, Inc.) and purified by Exo-SAP-IT PCR product clean-up reagent (Applied Biosystems, ThermoFisher) before sequencing. Sanger sequencing of the amplified DNA was carried out by FASMAC Co., Ltd (Kanagawa, Japan). Internal primers as listed in [14] were used to obtain full lengths of the 28S and 18S fragments. The electropherograms obtained were checked by eye on the software Geneious Prime 2022.2.2 (https://www.geneious.com/), the fragments of each gene were assembled and the consensus sequences were used for downstream phylogenetic analyses. Newly generated sequences were deposited on NCBI GenBank, accession numbers OP628171 and OP620447-OP620450 (electronic supplementary material, table S1).

### (c) Phylogenetic analyses

The newly obtained sequences were used together with GenBank sequences of other barnacles used in a previous study [7] plus four further poecillasmatid barnacles *Octolasmis hawaiense*, *Octolasmis unguisiformis*, *Oxynaspis ryukyuensis,* and *Rugilepas pearsei* (electronic supplementary material, table S2). The sequences were aligned by MUSCLE [15] implemented in Geneious Prime 2022.2.2 gene by gene, informative regions were then selected by Gblocks [16] with the options ‘allow smaller final blocks’ and ‘allow gap positions within the final blocks' selected, then concatenated for the downstream analyses. The final alignment was 4056 bp and included 102 barnacle species. Maximum-likelihood tree with the substitution model of TIM1 + I + G, selected as a suitable model by jModelTest 2 [17], was reconstructed by PhyML 3.0 [18] with 2000 bootstrap replicates and Bayesian inferences with MrBayes 3.2 [19], both implemented in Geneious Prime 2022.2.2. Results of the PhyML maximum-likelihood and Bayesian trees are shown in electronic supplementary material, figure S1.

**Figure 2.**
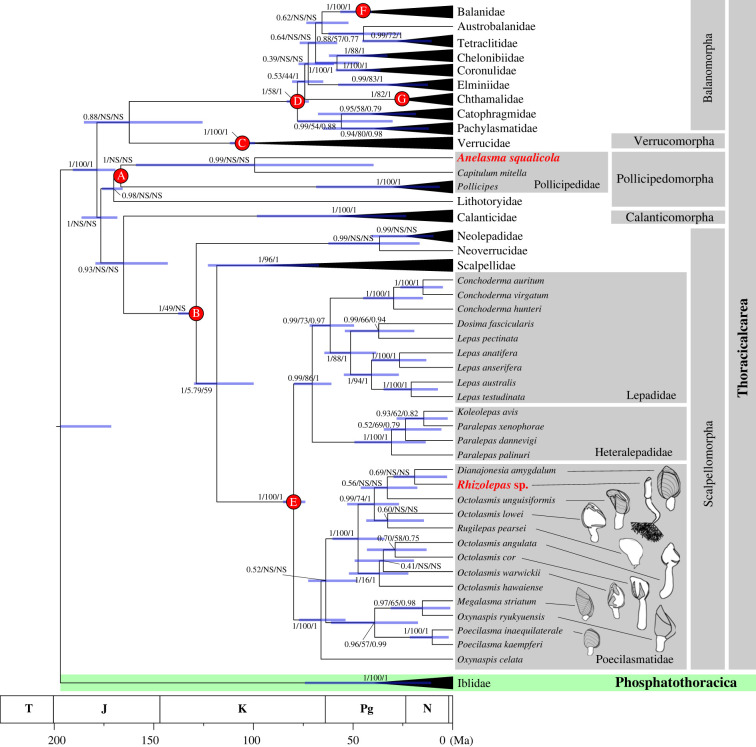
Phylogenetic reconstruction of barnacles using COI, 16S rRNA, 18S rRNA, and 28S rRNA showing Bayesian divergence-time estimates (node bars indicating 95% HPD confidence intervals) from BEAST. Node values indicate branch support in this order: posterior probability for maximum clade credibility/bootstrap value for maximum likelihood/Bayesian posterior probability. NS indicates a branch was not supported by that analysis. Drawings indicate the level of plate calcification. Red dots refer to fossil calibration points (alphabet letter codes corresponds to table 1).

Divergence ages of the clades were estimated by BEAST 2.7.3 [20]. Optimized relaxed clock and Yule models were applied with eight fossil calibration points as prior settings (table 1) according to [21–32] shown in figure 2. Details of input into BEAST for the fossil calibrations can be found in electronic supplementary material, table S3. The input alignment was partitioned by genes and for COI also by codon positions, with evolutionary models selected using jModelTest 2 for each partition. This selected HKY + I + G for 16S and TIM3 + I + G for all other partitions, but as TIM3 is unavailable in BEAST, GTR was used instead. The chain length of the run was 100 million generations, and the parameters were sampled every thousand generations. The logs were checked with Tracer v. 1.7.2 [33] and the convergence of the run were confirmed by effective sample size (ESS) higher than 300. The maximum credibility clade (MCC) tree, as well as divergence age estimations, were analysed by Tree Annotator after 25% burn-in process. The MCC tree was visualized by FigTree v. 1.4.4 (http://tree.bio.ed.ac.uk/software/figtree/) with the 95% highest posterior density (HPD) displayed.

**Table 1.** List of fossil records of barnacle clades used as calibration points in the present study. CI: confidence interval.

calibration	clade	origin	calibration fossil	input (Ma)		output (Ma)			references
				mean	97.5% CI	mean	5.00%	95.00%	
A	Pollicipedomorpha	Bathonian, Middle Jurassic (165.3–168.2 Ma)	*Concinnalepas bessiensis* †	167	166–168	166.70	165.4	168.02	[21–23]
B	Scalpellomorpha	Hauterivian, Lower Cretaceous (125.77–132.6 Ma)	*Jaegerscalpellum elegans* †	129	126–132	128.7	125.7	131.91	[24]
C	Verrucomopha	Albian, Lower Cretaceous (100.5–113 Ma)	*Eoverruca aubensis* †	107	100–113	105.7	99.22	111.97	[25]
D	Balanomorpha	Campanian, Upper Cretaceous (72.1–83.6 Ma)	*Pachydiadema cretaceum* †	77.7	72.1–83.6	78.08	72.62	83.712	[23,26]
E	Lepadoidea	Campanian, Upper Cretaceous (72.1–83.6 Ma)	*Poecilasma*? sp. *sensu* [27]	77.7	72.1–83.6	80.2	74.44	85.684	[27]
F	Balanidae	Lutetian - Bartonian (Bortonian), Eocene (39.1–42.6 Ma)	*Palaeobalanus lindsayi* †	40.8	39.1–42.6	41.01	39.3	42.776	[28]
G	Chthamalidae	Chattian (Duntroonian), Oligocene (25.2–27.3 Ma)	*Chamaesipho grebneffi* †	26.3	25.2–27.3	26.52	25.5	27.576	[29]

Additionally, IQ-TREE v. 2.2.0 [34] was used to reconstruct a maximum-likelihood phylogeny with input alignment partitioned by genes and also codon positions for COI, as for the BEAST analysis above. The IQ-TREE model finder was used to select suitable evolutionary models for each partition, which were TN + F + I + I + R3 for 28S, TIM2e + R3 for 18S, TVM + F + I + I + R4 for 16S, TIM2e + I + G4 for the first codon position of COI, TNe + I + G4 for the second codon position of COI, and TPM3 + I + G4 for the third codon position of COI.

The raw output file of all phylogenetic reconstructions, as well as alignment files before and after Gblock treatment, are available on Figshare (https://figshare.com/s/d75edd4adc42ad07729c) [35].

## 3. Results and discussion

Surprisingly, our four-gene phylogenetic reconstruction (figure 2; electronic supplementary material, figure S2) revealed *Rhizolepas* sp. nesting within the poecilasmatid genus *Octolasmis*. This resonates with a recent study of *Rugilepas*, another perplexing barnacle inducing gall-formation on urchins [11]. *Rugilepas* was originally assigned to the family Microlepadidae, but phylogenetics showed that it too was nested within *Octolasmis* [11]. Our phylogeny with an additional 16S rRNA sequence of *Rugilepas* further confirmed this (figure 2). Similarly, *Dianajonesia* is an epibiont of various animals which, as previously noted [11], also clustered with the foregoing taxa. As such, *Rhizolepas, Rugilepas*, and *Dianajonesia* are interpreted as derived members of *Octolasmis* and therefore synonymous with the latter at the genus level. This broadening of the taxon was recovered as a monophyletic clade with maximum support (figure 2).

Divergence date estimates suggest *Rhizolepas* diverged from *Dianajonesia* around 19.15 mya (95% confidence interval 2.88–38.86 Ma), while *Rugilepas* split from *O. lowei* around 32.94 (16.87–43.55) mya. Our phylogenies suggest *Oxynaspis* to be the likely sister genus of *Octolasmis*, as it was recovered as monophyletic and sister to *Octolasmis* in the IQ-TREE reconstruction (electronic supplementary material, figure S2), with a good support (SH-aLRT and ultrafast bootstrap support of 91.4 and 82, respectively). However, it was not monophyletic in the time-calibrated tree or the PhyML/Bayesian reconstructions, with *Oxynaspis ryukyuensis* nested within *Poecilasma*. Based on our phylogenetic reconstructions Rhizolepadidae, like Microlepadidae [4], is here synonymized with Poecilasmatidae. All members of Poecilasmatidae, recovered as a fully supported clade (figure 2) live on the body surface of animals [4], while most *Octolasmis* and *Dianajonesia* species infest decapods [36], *O. weberi* lives on cnidarians [37], *O. warwickii* occasionally infests molluscs, and *O. grayii* is known exclusively from sea snakes [38]. The addition of *Rhizolepas* and *Rugilepas* means this genus-level clade displays an array of transitional stages. It has been suggested that further enigmatic epibiotic or parasitic barnacles lacking molecular data, such as *Arcalepas* and *Malacolepas* infesting bivalves [36], may belong here too [4].

We propose that the epibiotic members of *Octolasmis*, which retain feather-like cirri for suspension-feeding, represent the first stage in the transition to parasitism where they begin to exploit hosts as commensals. Different species of *Octolasmis* exhibit varying levels of plate reduction linked to diverse degrees of host protection and association [11], where those with more reduced plates tend to correlate with increased host intimacy (degree of calcification shown as schematics on figure 2). This is indicative that the trend of increased plate reduction and host intimacy coincides with progression towards parasitism. *Dianajonesia* is also at the initial epibiotic stage whereas *Rugilepas* represents an intermediate stage with atrophied plates and cirri and moving from cementing to anchoring which increases the intimacy of the host–barnacle association [11]. In *Rugilepas*, the gall formed by the urchin appears to prevent the exploitation of host tissue; it instead feeds on particulate organic matter [11]. Lastly, *Rhizolepas* has penetrated the host defence and feeds on the host, evolving a root system for efficient exploitation. Although independently evolved, *Anelasma* likely went through similar transitions. The roots of both *Rhizolepas* and *Anelasma* show convergence with the ‘interna’ network of rhizocephalans [36], implying the final step is losing the peduncle and capitulum entirely. Outside the roots, *Rhizolepas* has nearly lost its plates completely and the body is largely occupied by gonads [6], suggesting the beginning of this step. Penetration of the host at the settlement larval stage is another key morphological adaptation in cirripede parasitism, with cypris larvae of coral-associated thoracicans having spear-shaped antennules and kentrogon larvae of rhizocephalans developing a hollow stylet for this purpose [39,40]. Though the cypris larvae of *Rhizolepas* remains unknown, it likely exhibits similar, convergent adaptation.

Settling on a wide array of substrata is a forte of barnacles [4], and the number of animal phyla used for this purpose by *Octolasmis* is remarkable, comprising at least six phyla including Annelida, Arthropoda, Chordata, Cnidaria, Echinodermata, and Mollusca. Considering that most genera or families of parasitic animals specialise in one host group at each life stage, perhaps such exploration of, and experimentation with, heterogeneous hosts by repeated host-switching promotes and drives the opportunity for a rare macroevolutionary novelty where parasitism is established, like in *Rhizolepas* and *Anelasma*. While the origin of *Octolasmis* is estimated to be Eocene at 47.58 mya (34.25–60.42 mya), *Rhizolepas* is Miocene in age at 19.15 mya (2.88–38.86 mya) and thus much more recent than *Anelasma*. The latter is estimated to be mid-Cretaceous in origin around 99.46 mya in the present study (39.39–160.01 mya), which is in agreement with a previous study, which also placed it in the mid-Cretaceous at 126.50 mya [11]*.* Although *Octolasmis* provides an exceptional glimpse at a stepwise transition from commensalism to parasitism, a similar pathway suggested for other parasitic lineages [41] is difficult to verify because many parasites are poorly preserved in the fossil record.

## Data Availability

Newly generated genetic data are deposited in NCBI GenBank under accession numbers OP628171 and OP620447-OP620450. Original alignments (before and after Gblocks trimming) and raw output files of all phylogenetic analyses are available from the Figshare repository: https://doi.org/10.6084/m9.figshare.21314334 [35]. Other data are provided in the electronic supplementary material [42].
